# Toledo Medical Association

**Published:** 1879-04

**Authors:** 


### TOLEDO MEDICAL ASSOCIATION.

The two hundred and forty-third regular meeting was held July 12, 1878.

Dr. Collamore presented the report of the committee on counter-prescribing by druggists.

Voted that the report be published in the city papers, and a copy be sent to each druggist in the city, with an invitation to be present at the next meeting of the Association.

The two hundred and forty-fourth regular meeting was held July 26, 1878.

*Counter-Prescribing.*—Ten druggists, or their representatives, were present. Dr. Collamore stated the resolutions adopted by the Association.

Mr. C. Hohly, druggist, asked if any of the gentlemen knew of any case where the druggist had offered his services. The people ask for them, and it is hard to turn them off. Would plead guilty to the charge of prescribing, but what could the druggist do?

Dr. Thorn remarked that there was no personal feeling in the matter, and that the people were the sufferers; that the druggist will be allowed to go as far as the physician—that is, as far as the law will allow. In order to practice medicine lawfully, it is necessary to have a diploma or ten years' practice.

Dr. Cherry considered it legitimate for druggists to prescribe in a certain way—cough mixture, etc.

Dr. Chapman remarked that he knew from experience that druggists could not help prescribing in certain cases, but that they must assume the responsibility.

Mr. A. Gates, druggist, did not believe that there was as much counter-prescribing as the doctors thought.

Mr. Cheney, druggist, did not think that any first-class druggist would be willing to take the responsibility of prescribing for any complicated case. Cannot help prescribing for trivial effections.

Mr. Burger, druggist, remarked that the people expect the druggist to know something about these small diseases. If I don't prescribe for them somebody else will, and thus I lose my trade.

Mr. J. Gates and Mr. I. N. Reed claimed to do no prescribing.

Dr. Cullison, druggist, always sends such applicants to a doctor, if possible; never prescribes when he can possibly avoid it.

Dr. Reed said it was a right we all have to send our prescriptions to any particular druggist. Each druggist has a right to recommend any physician he prefers.

Dr. Waddel remarked, that the reason that the people go to the druggist is, that they get the advice and medicine for the price of the medicine alone. When a druggist will detail symptoms and put up prescriptions, and charge only for the medicine, it is simply robbing the doctors. Wouldn't find fault with the druggists if they would charge for both.

Dr. Ridenour said that when the druggists examine a patient and prescribe, charging only for the medicine, they enter into direct competition with the doctors. Can the druggist afford this, and will the doctors submit to it? Will not the doctors furnish their own medicines, and thus starve out half the drug stores?

Dr. Kirkley didn't think that it injured the doctors at all; it really increased their business. The druggists and the patients were the sufferers. The matter resolves itself into a question of ethics: The duties of druggists to physicians, and of physicians to druggists.—*Toledo Medical and Surgical Journal.*

